# Inhibition of melanin production by anthracenone dimer glycosides isolated from *Cassia auriculata* seeds

**DOI:** 10.1007/s11418-018-01276-2

**Published:** 2019-03-07

**Authors:** Weicheng Wang, Yi Zhang, Souichi Nakashima, Seikou Nakamura, Tao Wang, Masayuki Yoshikawa, Hisashi Matsuda

**Affiliations:** 1 Kyoto Pharmaceutical University, Misasagi, Yamashina-ku, Kyoto, 607-8412 Japan; 2 Tianjin State Key Laboratory of Modern Chinese Medicine, Tianjin University of Traditional Chinese Medicine, 312 Anshanxi Road, Nankai District, Tianjin, 300193 China; 3 N.T.H Co., Ltd., 4F Sky-ebisu Bldg, 1-8-11 Ebisu, Shibuya-ku, Tokyo, 150-0013 Japan

**Keywords:** *Cassia auriculate*, Anthracenone dimer glycoside, Melanogenesis inhibitor, Mechanism of action

## Abstract

The methanol extract of *Cassia auriculata* seeds was found to inhibit melanogenesis in B16 melanoma 4A5 cells under conditions of theophylline stimulation. Two new phlegmacin-type anthracenone dimer glycosides, auriculataosides A and B, were isolated from the active methanol fraction, and their inhibitory effects were observed in the concentration range of 0.03 to 0.3 μM. Inhibition of microphthalmia-associated transcription factor, tyrosinase, tyrosinase-related protein (TRP)-1, and TRP-2 protein expression was observed, suggesting that the inhibition of these factors is part of the mechanism of action underlying melanogenesis inhibition.

**Electronic supplementary material:**

The online version of this article (10.1007/s11418-018-01276-2) contains supplementary material, which is available to authorized users.

## Introduction

Melanocytes originate from the ganglion in the embryonic phase and are distributed in the epidermal basal layer, hair follicles, uvea of the eye, soft brain membrane, oral mucosa, inner ear, peritoneal membrane, and eye socket. The formation of cutaneous melanin is a normal physiological phenomenon that plays an important role in protecting skin from UV injury [1, 2]. However, melanin production can be promoted—for example, by lifestyle choices, long-term use of the psychotic drug chlorpromazine and the anti-epileptic drug phenytoin sodium, among others [3]. Excessive melanin deposition causes such pigmentary disorders such as melasma, spots, and senile pigment spots [4]. In addition, drugs such as chlorpromazine, streptomycin, chloroquine, and phenothiazine tend to electrostatically bind to melanin easily and accumulate in the body, possibly causing side effects [5–7]. Inhibitors of melanin production are available, but those currently in use do not provide optimum protection because they induce side effects such as allergies and damage the stratum corneum, etc. Clearly, a melanin production inhibitor that has stronger effects and fewer side effects is desired. Our group has been searching for melanogenesis inhibitors in theophylline-stimulated B16 melanoma 4A5 cells treated with natural medicines and have reported various melanin production inhibitors, such as diarylheptanoids; flavonoids; triterpene saponins; benzylisoquinoline, carbazole, and pyrrolidinoindoline alkaloids; and lignan dicarboxylates [8–20].

The leguminous plant *Cassia auriculata* is a perennial evergreen shrub that is native to India, Sri Lanka, and other parts of Asia. The flower, leaves, stem, root, and unripe fruit of this shrub have been used for the treatment of diabetes, conjunctivitis, and skin diseases in Ayurveda, Indian traditional medicine. We have reported the isolation of various active compounds, including benzocoumarins, flavones, flavonols, and a flavanol dimer, with hepatoprotective effects in vitro from the leaves of *C. auriculate* [21]. However, little research has been done on the seed components of this plant and their biological activities [22].

In the study reported here we found that the methanol (MeOH) extract of *C. auriculata* seeds inhibited melanogenesis. We used bioassay-guided isolation techniques and further isolated two new anthracenone dimer glycosides, auriculataosides A (**1**) and B (**2**), as the active compounds. The isolation and chemical elucidation of **1** and **2** as well as their modes of action are also discussed herein.

## Results and discussion

### Extraction and isolation of glycosides** 1**–**4** from the seeds of *C. auriculata*

**Fig. 1:**
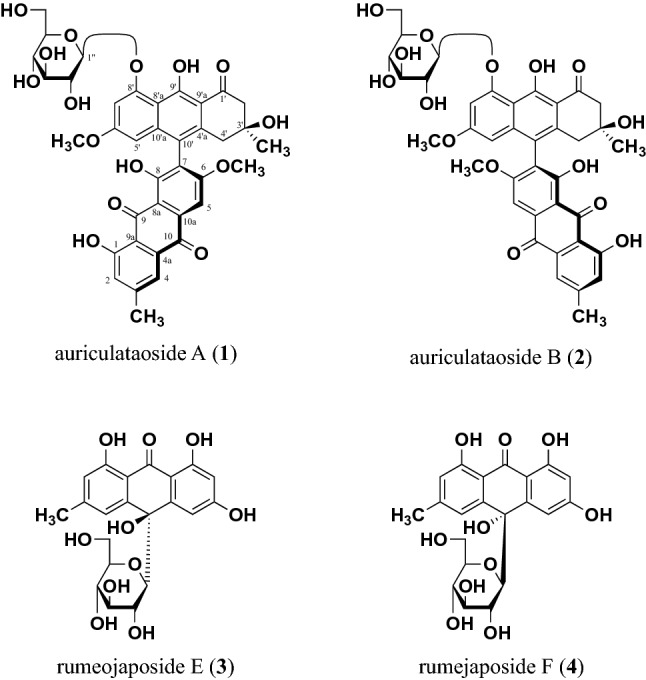
Chemical structures of anthracenone glycosides **1**–**4**

Seeds of *C. auriculata* (3929 g) were extracted with MeOH three times under reflux to yield a MeOH extract (430.6 g, yield: 10.96%). To defat the MeOH extract, part (399.0 g) of the extract was suspended in H_2_O and extracted with *n*-hexane to give an *n*-hexane-soluble fraction (46.4 g, 1.27%) and a H_2_O-soluble fraction (352.6 g, 9.63%). Part of the H_2_O-soluble fraction (322.7 g) was subjected to Diaion HP-20 column chromatography and eluted sequentially, first with H_2_O and then with MeOH, to obtain the MeOH-eluted fraction (63.2 g, 1.89%) and H_2_O-eluted fraction (259.5 g, 7.41%). The MeOH-eluted fraction was repeatedly separated and purified using normal silica gel (CHCl_3_–MeOH) and reversed-phase (ODS) (MeOH–H_2_O) column chromatography and HPLC (YMC-Pack ODS-5-A, 250 × 20 mm i.d., MeOH–H_2_O or CH_3_CN–H_2_O). These efforts yielded two new anthracenone dimer glycosides named auriculataosides A (**1**, 0.011%) and B (**2**, 0.0084%) and known anthraquinone glycosides rumejaposides E (**3**, 0.00038%) and F (**4**, 0.00026%) [23] (Fig. 1).

### Structures of auriculataosides A (**1**) and B (**2**)

Auriculataosides A (**1**) and B (**2**) were obtained as red powder with negative optical rotation (**1**: [*α*]_D_^24^ − 129.5°; **2**: [*α*]_D_^22^ − 41.3° in 1,4-dioxane). FAB-MS measurement revealed a pseudo molecular ion peak at *m/z* 755 (M+Na)^+^, respectively, and the molecular formula of both compounds was determined as C_38_H_36_O_15_ by high-resolution FAB-MS measurement. Their IR absorption spectra showed absorptions (**1** and **2**: 3450, 1655, and 1070 cm^−1^) ascribable to hydroxy, carbonyl, and ether functions, respectively. The ^1^H-NMR (DMSO-*d*_6_) and the ^13^C-NMR spectra of **1** and **2** (Table 1), the signals of which were assigned based on the results of various NMR experiments, showed resonances assignable to two methyls [**1**: *δ* 1.15, 2.24 (3H each, both s, C*H*_3_-3′, 3); **2**: *δ* 1.18, 2.24 (3H each, both s, C*H*_3_-3′, 3)], two methylenes [**1**: δ 2.61 (2H, br s, H_2_-4′), 2.74, 2.88 (1H each, both d, *J  *= 16.8 Hz, H_2_-2′); **2**: *δ* 2.57, 2.75 (1H each, both d, *J* = 16.2 Hz, H_2_-4′), 2.72, 2.90 (1H each, both d, *J  *= 16.8 Hz, H_2_-2′)], two methoxy groups [**1**: *δ* 3.65, 3.89 (3H each, both s, OC*H*_3_-6′, 6); **2**: *δ* 3.66, 3.85 (3H each, both s, OC*H*_3_-6′, 6)], five aromatic protons [**1**: *δ* 6.20, 6.88 (1H each, both d, *J * = 2.1 Hz, H-5′, 7′), 7.22, 7.59 (1H each, both br s, H-2, 4), 7.55 (1H, s, H-5); **2**: *δ* 6.20, 6.89 (1H each, br s, H-5′, 7′), 7.18, 7.53 (1H each, both br s, H-2, 4), 7.52 (1H, s, H-5)], and a β-d-glucopyranosyl moiety [**1**: δ 5.04 (1H, d, *J * = 7.7 Hz, H-1″); **2**: *δ* 5.02 (1H, d, *J  *= 7.7 Hz, H-1″)].

**Table 1:** ^13^C-NMR spectroscopic data for anthracenone dimer glycosides auriculataosides A (**1**) and B (**2**) in DMSO-*d*_6_

Position	**1**	**2**	Position	**1**	**2**	Position	**1**	**2**
1	161.4	161.4	1′	204.1	204.2	1″	101.6	101.8
2	124.2	124.2	2′	51.6	51.4	2″	73.4	73.4
3	148.6	148.6	3′	68.8	68.8	3″	76.4	76.4
4	120.6	120.6	4′	41.2	41.0	4″	69.8	69.8
4a	132.8	132.6	4′a	136.6	136.7	5″	77.3	77.3
5	103.2	103.3	5′	99.6	99.4	6″	60.8	60.8
6	163.8	163.8	6′	161.4	161.4	*C*H_3_-3	21.5	21.5
7	120.1	120.1	7′	100.7	100.8	*C*H_3_-3′	28.2	28.5
8	161.1	161.2	8′	159.2	159.3	O*C*H_3_-6	56.8	56.5
8a	110.7	110.7	8′a	109.6	109.6	O*C*H_3_-6′	55.1	55.1
9	190.5	190.5	9′	164.7	164.6			
9a	113.4	113.4	9′a	109.4	109.4			
10	181.3	181.2	10′	117.1	117.1			
10a	134.6	134.5	10′a	139.4	139.4			

**Fig. 2:**
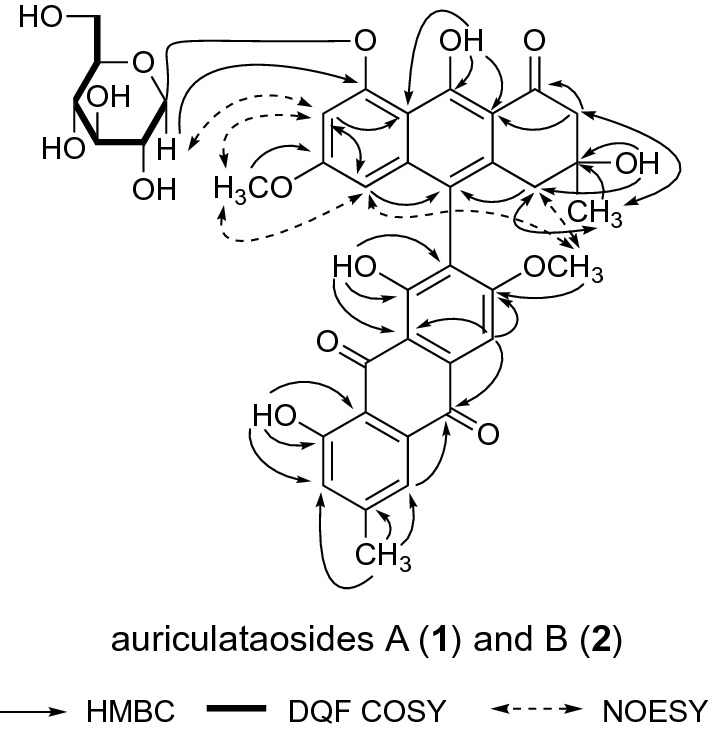
Important two dimensional-NMR correlations of auriculataosides A (**1**) and B (**2**).* DQF *Double Quantum Filter

The COSY-Double Quantum Filter and HMBC experiments revealed both **1** and **2** to be anthracenone derivatives with a glucopyranosyl moiety at position 8. Acid hydrolysis of **1** and **2** with 1.0 M HCl yielded d-glucose, which was identified by HPLC equipped with an optical rotation detector. In the HMBC experiments, ^1^H-^13^C long-range correlations were observed between the following proton–carbon pairs (H-4 and C-10; H-5 and C-6, 8a, 10; H-2′ and C-1′, 9′a, *C*H_3_-3; H-4′ and C-10′, *C*H_3_-3′; H-5′ and C-6′, 7′, 10′; H-7′ and C-5′, 8′, 8′a; H-1″ and C-8′; C*H*_3_-3 and C-2, 3, 4; C*H*_3_-3′ and C-2′, 3′, 4′; O*H*-1 and C-1, 2, 9a; O*H*-8 and C-7, 8, 8a; O*H*-3′ and C-3′, 4′, *C*H_3_-3′; O*H*-9′ and C-8′a, 9′, 9′a; OC*H*_3_-6 and C-6; OC*H*_3_-6′ and C-6′) (Fig. 2). NOESY experiments on **1** and **2** showed correlations between the following proton–proton pairs (H-1″ and H-7′; C*H*_3_-3 and H-2, 4; OC*H*_3_-6 and H-4′, 5, 5′; OC*H*_3_-6′ and H-5′, 7′) (Fig. 2). These results showed that **1** and **2** are phlegmacin-type anthracenone dimers in which C-7 is connected to C-10′ and that both **1** and **2** are mutually atropisomers, as shown.

The absolute configurations of the asymmetric axes in **1** and **2** were determined by comparing their CD spectra with those of known anthracenone dimers. Elsworth et al. reported that phlegmacin-type anthracenone dimer (3′*S*,*P*)-anhydrophlegmacin-9,10-quinone 8′-*O*-methyl ether [**S1**; see Electronic Supplementary Material (ESM) Fig. S1] having the *S* configuration at the 7–10′ stereostructure exhibited a positive Cotton effect (*Δε* + 22.6) at 267 nm and a negative Cotton effect (*Δε* − 34.4) at 283 nm [24]. The Cotton effects of **S1** centered around 275 nm is in agreement with the anticlockwise twist between the napthalenoid and anthraquinone rings. By subsequent application of the Prelog–Helmchen rules [25], these authors determined the axial configurations of **S1** [24]. As shown in ESM Fig. S2, **2** showed the very similar CD spectrum of a positive Cotton effect (*Δε* + 28.3) at 270 nm and a negative Cotton effect (*Δε* − 27.9) at 288 nm; its absolute configuration was determined to be *S*. To the contrary, as **1** showed the opposite axial configuration to **2** based on a negative Cotton effect (*Δε* − 38.0) at 270 nm and a positive Cotton effect (*Δε* + 25.5) at 284 nm, its absolute configuration was determined to be *R* at the 7–10′ stereostructure.

With regard to the stereostructure at the 3′-position, Elsworth et al. also summarized that the comparison of the chemical shift and coupling constants of 4′-H_ax_ and 4′-H_eq_ with the corresponding data for other phlegmacins revealed an empirical relationship between the difference (*Δδ*) in the chemical shift of these methylene protons and the relative configuration between the C-3′ stereostructure and biaryl axis [24]. Thus, when the C-3′ hydroxy group is on the same side of the C-10′-anthracenone (anthraquinone) ring (′syn′), chemical shift differences (Δδ) between the 4′-H_ax_ and 4′-H_eq_ trend to be large (*Δδ  *= 0.15–0.25). However, when the C-3′ hydroxy group and the C-10′-anthracenone (anthraquinone) ring are on apposite sides of the molecules (‘anti’), *Δδ* are often much smaller (*Δδ* ≤ 0.08 ppm). In ^1^H-NMR spectra, the Δδ between 4′-H_ax_ and 4′-H_eq_ of **1** were the same value (0 ppm), and those of **2** were 0.18 ppm, suggesting that the configurations at the 3′-position of **1** and **2** were *S*. Thus, the chemical structures of auriculataosides A (**1**) and B (**2**) were determined as shown.

Known compounds **3** and **4** were identified by comparison of their physical data ([α]_D_,^1^H- and ^13^C-NMR, and MS spectra) with reported values [23].

### Effects on melanogenesis in B16 melanoma 4A5 cells

Melanocytes are stimulated by many effectors, including UV radiation and α-melanocyte-stimulating hormone (α-MSH) [26, 27]. It is generally accepted that the cAMP pathway plays a key role in the regulation of melanogenesis and that cAMP is involved in α-MSH-stimulated signal transduction [28, 29]. B16 melanoma cells have often been used in experiments of melanogenesis stimulated by α-MSH or phosphodiesterase inhibitors. Here, we used phosphodiesterase inhibitor theophylline to stimulate B16 melanoma 4A5 cells.

As shown in Table 2, the MeOH extract and the MeOH-eluted fraction exhibited significant inhibition on melanogenesis in the concentration range of 1–100 µg/mL, and the MeOH-eluted fraction exhibited cytotoxicity at 100 µg/mL. As the MeOH-eluted fraction exhibited inhibitory effects on melanogenesis in these cells, the effects of **1**–**4**, which are components of the MeOH-eluted fraction, on melanogenesis were examined. Compounds **1** and **2** showed significantly stronger melanogenesis inhibitory effects (45.1% and 36.5% inhibition, respectively, at 0.3 μM) than the reference compound β-arbutin (32.1% inhibition at 100 µM) (Table 3), and both compounds showed cytotoxicity at 1 μM (Table 3). Compounds **3** and **4** showed weaker melanogenesis inhibitory effects and lower cytotoxicity than did compounds **1** and **2**; however, **1** and **2** did not show cytotoxicity toward human dermal fibroblasts (HDF) at 0.01–1 µM (Table 4), suggesting that they selectively inhibited the proliferation of melanoma cells.

**Table 2:** Inhibitory effects of MeOH extract and its fractions from the seeds of *C. auriculata* at different concentrations on melanogenesis and cell viability in B16 melanoma 4A5 cells

Sample	Inhibition (%)						IC_50_ (μg/mL)
	0 µg/mL	1 µg/mL	3 µg/mL	10 µg/mL	30 µg/mL	100 µg/mL	
MeOH extract	0.0 ± 1.3	29.2 ± 2.9**	28.7 ± 2.6**	32.0 ± 1.7**	35.1 ± 2.7**	36.1 ± 3.7**	–
MeOH-eluted fraction	0.0 ± 2.7	− 2.0 ± 3.6	7.2 ± 4.2	22.2 ± 4.6**	27.8 ± 1.5**	–	–
H_2_O-eluted fraction	0.0 ± 4.9	5.3 ± 3.1	8.9 ± 3.0	10.5 ± 5.9	13.5 ± 4.9	27.7 ± 1.8**	–

Sample	Cell viability (%)						IC_50_ (μg/mL)
	0 µg/mL	1 µg/mL	3 µg/mL	10 µg/mL	30 µg/mL	100 µg/mL	
MeOH extract	100.0 ± 2.5	106.0 ± 2.2	103.1 ± 2.1	96.6 ± 1.8	96.9 ± 3.2	107.8 ± 3.4	–
MeOH-eluted fraction	100.0 ± 3.6	95.1 ± 3.4	98.5 ± 2.8	96.7 ± 1.5	80.1 ± 0.6**	42.8 ± 1.3**	80.5
H_2_O-eluted fraction	100.0 ± 4.1	97.6 ± 3.6	101.0 ± 0.6	102.5 ± 1.0	97.2 ± 3.8	101.6 ± 2.7	–

Each value represents the mean ± standard error of the mean (SEM) (*N *= 4)

**Significantly different from the control group at *p* < 0.01

**Table 3:** Inhibitory effects of anthracenone glycosides **1**–**4** and β-arbutin at different concentrations on melanogenesis and cell viability in B16 melanoma 4A5 cells

Sample	Inhibition (%)						IC_50_ (μM)
	0 μM	0.01 μM	0.03 μM	0.1 μM	0.3 μM	1 μM	
**1**	0.0 ± 4.0	17.5 ± 3.4**	21.5 ± 2.7**	31.9 ± 2.1**	45.1 ± 0.4**	–	–
**2**	0.0 ± 4.1	8.8 ± 4.0	17.2 ± 4.0*	19.6 ± 5.3*	36.5 ± 3.3**	–	–
**3**	0.0 ± 1.1	15.4 ± 0.6**	13.2 ± 3.9*	21.2 ± 3.8**	22.0 ± 2.2**	24.7 ± 1.1**	–
**4**	0.0 ± 3.9	12.2 ± 4.5	16.4 ± 3.4*	15.9 ± 1.9*	8.5 ± 3.5	16.8 ± 2.4**	–

Sample	Inhibition (%)						IC_50_ (μM)
	0 μM	10 μM	30 μM	100 μM	300 μM	1000 μM	
β-Arbutin	0.0 ± 1.9	12.5 ± 1.4	12.3 ± 4.7	32.1 ± 2.4**	53.2 ± 3.3**	–	262

Sample	Cell viability (%)						IC_50_ (μM)
	0 μM	0.01 μM	0.03 μM	0.1 μM	0.3 μM	1 μM	
**1**	100.0 ± 0.8	101.8 ± 1.9	101.8 ± 1.7	104.7 ± 1.9	94.7 ± 3.2	27.0 ± 2.1**	0.75
**2**	100.0 ± 1.8	104.6 ± 1.3	103.3 ± 1.1	100.4 ± 2.7	91.9 ± 1.1*	29.0 ± 1.4**	0.75
**3**	100.0 ± 1.0	104.5 ± 0.9	102.6 ± 0.8	103.5 ± 0.8	102.4 ± 0.8	99.5 ± 0.8	–
**4**	100.0 ± 1.3	102.0 ± 1.1	104.0 ± 3.6	102.1 ± 2.0	101.1 ± 1.1	100.5 ± 1.0	–

Sample	Cell viability (%)						IC_50_ (μM)
	0 μM	10 μM	30 μM	100 μM	300 μM	1000 μM	
β-Arbutin	100.0 ± 2.2	93.5 ± 2.3	86.9 ± 2.8	87.9 ± 2.6	81.1 ± 1.2	46.1 ± 2.2**	902

Each value represents the mean ± SEM (*N * = 4)

*, **Significantly different from the control group at *p < 0.05 and **p < 0.01

**Table 4:** Effects of auriculataosides A (**1**) and B (**2**) at different concentrations on cell viability in human dermal fibroblasts

Sample	Cell viability (%)						IC_50_ (μM)
	0 μM	0.01 μM	0.03 μM	0.1 μM	0.3 μM	1 μM	
**1**	100.0 ± 6.7	99.7 ± 6.4	99.7 ± 5.6	92.0 ± 3.3	95.6 ± 6.6	93.8 ± 6.2	–
**2**	100.0 ± 5.0	96.0 ± 4.0	93.7 ± 3.9	97.0 ± 1.6	92.9 ± 3.8	95.3 ± 3.9	–

Each value represents the mean ± SEM (*N  *= 4)

### Effects of anthracenone glycosides **1**–**4** on tyrosinase

In human and mouse, melanin is produced from tyrosine as the starting material by the oxidation and polymerization of dopachrome produced by such enzymes as tyrosinase and tyrosinase-related protein (TRP)-1, and TRP-2. Therefore, to investigate the mechanism of action underlying melanogenesis inhibition, we examined the inhibition of tyrosinase, the main melanin synthetase, using mushroom- or melanoma-derived tyrosinase. Kojic acid and β-arbutin [8, 9] were used as reference compounds.

Mushroom-derived tyrosinase has been conventionally used for the development of tyrosinase inhibitors [8, 9]. In order to investigate the mechanism of action underlying melanogenesis inhibition, we examined the inhibitory effects of anthracenone glycosides **1**–**4 **on mushroom-derived tyrosinase in the concentration range of 0.01 to 100 μM. No significant inhibitory effect was found for auriculataosides A (**1**) and B (**2**) even at the high concentration of 100 µM (Table 5).

**Table 5:** Effects of anthracenone glycosides **1**–**4**, kojic acid, and β-arbutin at different concentrations on mushroom tyrosinase activity

Sample	Inhibition (%)						IC_50_ (μM)
	0 μM	0.01 μM	0.1 μM	1 μM	10 μM	100 μM	
**1**	0.0 ± 2.7	− 3.5 ± 0.7	− 3.1 ± 2.0	− 2.0 ± 2.4	− 1.7 ± 2.6	− 2.2 ± 2.0	–
**2**	0.0 ± 2.9	− 2.2 ± 0.9	− 1.4 ± 1.5	0.5 ± 1.1	0.2 ± 0.9	0.2 ± 1.0	–
**3**	0.0 ± 0.8	− 1.1 ± 1.5	− 0.5 ± 1.6	− 0.1 ± 1.8	2.3 ± 2.9	3.2 ± 1.0	–
**4**	0.0 ± 2.4	− 3.0 ± 1.3	− 0.1 ± 1.6	0.4 ± 1.7	3.2 ± 3.7	2.2 ± 1.5	–

Sample	Inhibition (%)						IC_50_ (μM)
	0 μM	1 μM	3 μM	10 μM	30 μM	100 μM	
Kojic acid	0.0 ± 1.7	12.4 ± 4.9	20.8 ± 2.6**	41.1 ± 2.7**	63.7 ± 1.4**	85.4 ± 0.5**	15.5

Sample	Inhibition (%)						IC_50_ (μM)
	0 μM	10 μM	30 μM	100 μM	300 μM	1000 μM	
β-Arbutin	0.0 ± 1.3	1.3 ± 2.6	3.8 ± 1.2	6.6 ± 1.5*	10.2 ± 0.6**	20.6 ± 1.4**	–

Each value represents the mean ± SEM (*N  *= 4)

*, **Significantly different from the control group at *p < 0.05 and **p < 0.01

On the other hand, it has been reported that there is a considerable difference in the inhibitory effects of inhibitors on mushroom-derived tyrosinase and melanoma-derived tyrosinase [30–33]. Therefore, the inhibitory effect of the isolated anthracenone glycosides and reference compounds on melanoma-derived crude tyrosinase was investigated in the concentration range of 0.01 to 1 μM, which is the effective concentration range for melanogenesis. Kojic acid, a reference compound, exhibited a significant inhibitory effect on mushroom-derived tyrosinase but not on melanoma-derived tyrosinase. Compounds **1**–**4** did not inhibit the melanoma-derived tyrosinase at 1 µM (Table 6). These findings suggest that tyrosinase inhibition is not the main mechanism underlying melanin inhibition.

**Table 6:** Effects of anthracenone glycosides **1**–**4**, kojic acid, and β-arbutin at different concentrations on melanoma tyrosinase activity

Sample	Inhibition (%)						IC_50_ (μM)
	0 μM	0.01 μM	0.03 μM	0.1 μM	0.3 μM	1 μM	
**1**	0.0 ± 0.4	− 0.6 ± 0.6	− 2.7 ± 1.3	− 2.8 ± 1.0	− 0.1 ± 1.1	− 0.8 ± 1.3	–
**2**	0.0 ± 1.8	− 0.9 ± 0.8	− 0.3 ± 0.7	1.5 ± 0.5	− 2.2 ± 1.0	− 5.6 ± 0.6	–
**3**	0.0 ± 1.4	− 5.4 ± 1.2	− 8.1 ± 3.5	− 10.3 ± 0.6	− 9.5 ± 0.6	− 9.8 ± 0.8	–
**4**	0.0 ± 0.9	− 1.9 ± 1.7	− 1.3 ± 0.3	− 5.0 ± 0.7	− 2.5 ± 1.5	− 6.4 ± 2.4	–

Sample	Inhibition (%)						IC_50_ (μM)
	0 μM	1 μM	3 μM	10 μM	30 μM	100 μM	
Kojic acid	0.0 ± 2.0	− 2.0 ± 0.5	− 2.8 ± 0.7	− 1.0 ± 0.8	− 2.0 ± 1.8	− 10.9 ± 1.1	–

Sample	Inhibition (%)						IC_50_ (μM)
	0 μM	10 μM	30 μM	100 μM	300 μM	1000 μM	
β-Arbutin	0.0 ± 0.6	2.2 ± 1.2	6.1 ± 1.6	18.0 ± 0.7**	38.1 ± 0.9**	56.6 ± 0.4**	600

Each value represents the mean ± SEM (*N * = 4)

**Significantly different from the control group at *p* < 0.01

### Effects of anthracenone glycosides** 1**–**4** on autoxidation

In melanogenesis, dopachrome is synthesized from tyrosine through L-DOPA (l-3,4-dihydroxyphenylalanine) and dopaquinone by the action of an enzyme, such as tyrosinase. Produced dopachrome is automatically oxidized and polymerized into melanin [34]. Therefore, to investigate the mechanism of action underlying melanogenesis inhibition by compounds **1** and **2**, we examined the autoxidation inhibitory effects of **1** and **2** after dopachrome synthesis in the concentration range of 0.01 to 100 μM; ascorbic acid was used as the positive control [35]. Compounds **1** and **2** did not show significant inhibitory effects. On the contrary, they did show autoxidation-promoting effects at a high concentration (100 µM) (Table 7). Nevertheless, we believe that the color of the test sample may have affected the results at higher concentrations.

**Table 7:** Effects of anthracenone glycosides **1**–**4** and ascorbic acid at different concentrations on autoxidation

Sample	Inhibition (%)						IC_50_ (μM)
	0 μM	0.01 μM	0.1 μM	1 μM	10 μM	100 μM	
**1**	0.0 ± 5.8	− 3.1 ± 6.7	− 10.6 ± 6.6	6.9 ± 6.1	3.1 ± 5.8	− 36.8 ± 6.2**	–
**2**	0.0 ± 4.3	− 8.8 ± 6.2	− 13.7 ± 3.8	− 5.5 ± 7.2	− 24.5 ± 11.3	− 68.3 ± 3.9**	–
**3**	0.0 ± 10.6	− 3.7 ± 9.4	− 10.7 ± 11.2	− 13.3 ± 10.9	− 6.8 ± 9.0	− 10.4 ± 5.9	–
**4**	0.0 ± 11.2	3.5 ± 8.1	− 7.2 ± 11.7	− 22.1 ± 5.5	− 6.2 ± 5.3	− 17.2 ± 3.0	–

Sample	Inhibition (%)						IC_50_ (μM)
	0 μM	1 μM	3 μM	10 μM	30 μM	100 μM	
Ascorbic acid	0.0 ± 3.3	6.1 ± 0.6	15.0 ± 1.6	20.8 ± 1.7**	33.4 ± 2.8**	47.7 ± 1.8**	*ca*. 100

Each value represents the mean ± SEM (*N * = 4)

**Significantly different from the control group at *p  *< 0.01

### Effects of **1** and **2** on microphthalmia-associated transcription factor, tyrosinase, TRP-1, and TRP-2 protein expression levels

**Fig. 3:**
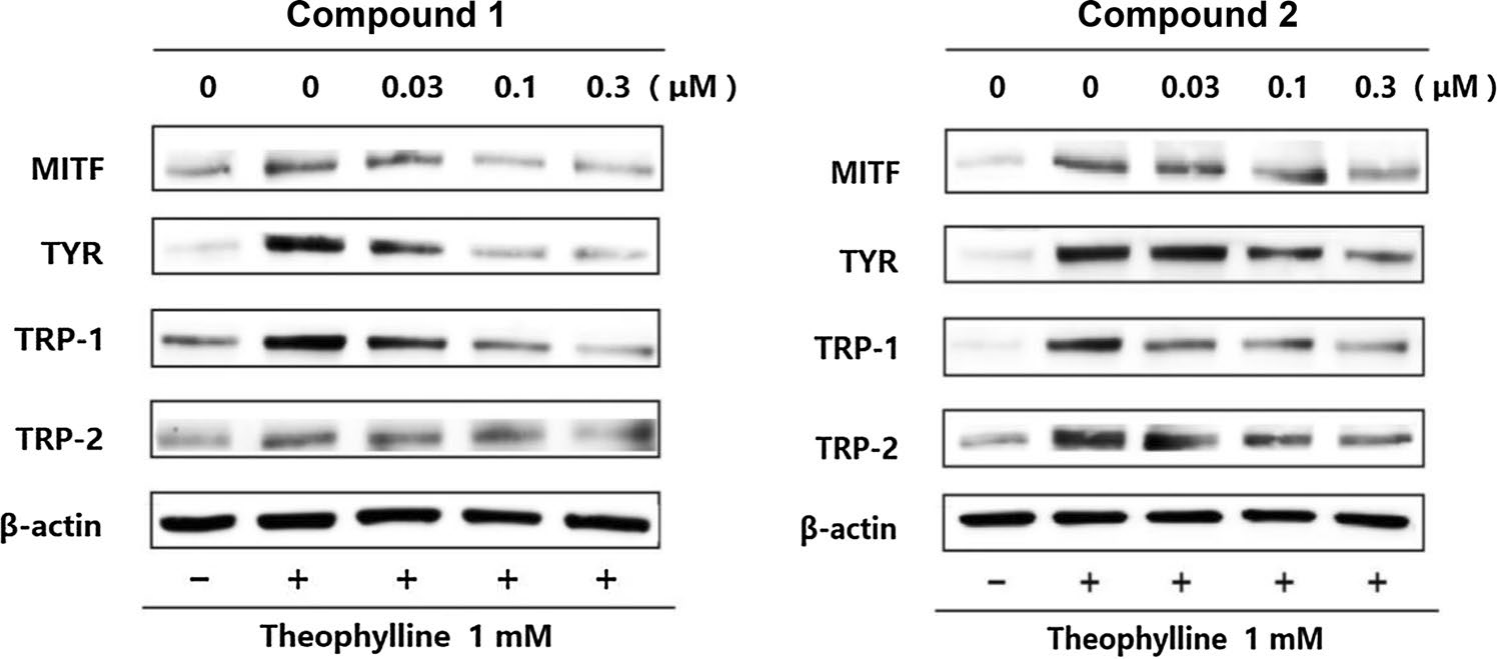
Effects of auriculataosides A (**1**) and B (**2**) on microphthalmia-associated transcription factor (MITF), tyrosinase (TYR) and tyrosinase-related protein (TRP)-1, and TRP-2 protein levels

In human and mouse, intracellular signal transduction is activated by increasing cAMP expression through UV irradiation or stimulation, such as α-MSH, which activates microphthalmia-associated transcription factor (MITF). mRNA transcription and translation of target proteins, such as tyrosinase, are promoted, and then melanin is produced [2]. Therefore, in order to investigate the mechanism of action underlying melanogenesis inhibition, we examined the effects of compounds **1** and **2** on the production of tyrosinase, MITF, TRP-1, and TRP-2, which are important proteins involved in melanin production, under theophylline-stimulated conditions in B16 melanoma 4A5 cells. β-Actin was used as the loading control. Expression levels of MITF, tyosinase, TRP-1, and TRP-2 after treatment with 0.3 µM of compounds **1** and **2** were 56 and 39%, 43 and 60%, 46 and 40%, and 48 and 37%, respectively. Namely, decreases in MITF, tyrosinase, TRP-1, and TRP-2 protein expression levels were observed after treatment with **1** and **2** in the concentration range of 0.1 to 0.3 μM (Fig. 3, ESM Fig. S3). These findings suggest that the inhibition of MITF, tyrosinase, TRP-1, and TRP-2 production is the main mechanism of action of **1** and **2**. Further studies are warranted to clarify their effects on the upper signaling pathway of MITF.

## Conclusions

Two new phlegmacin-type anthracenone dimer glycosides, here compounds **1** and **2**, isolated from the seeds of *C. auriculata* were examined for melanogenesis inhibitory effects in B16 melanoma 4A5 cells under theophylline-stimulated conditions. Compounds **1** and **2** significantly inhibited melanin production in the concentration range of 0.03 to 0.3 μM. We suggest the inhibition of MITF, tyrosinase, TRP-1, and TRP-2 protein expression is part of their mechanism of action.

## Materials and methods

### Instrumentation and material used to obtain physical data

Optical rotations were measured using a SEPA-300 digital polarimeter (*l* = 0.5) (Horiba Ltd., Kyoto, Japan). High-resolution mass spectrometry (high-resolution FAB-MS, EI-MS) and mass spectrometry (FAB-MS, EI-MS) were carried out using JMS-SX102 and JMS-GCMATE mass spectrometers (JEOL Ltd., Tokyo, Japan). The IR absorption spectrum was measured using a FT-IR DR-8000 spectrometer (Shimadzu Corp., Kyoto, Japan). The UV absorption spectrum was measured using a UV-1600 spectrometer (Shimadzu Corp.). The CD spectrum was measured using a J-1500 circular dichroism spectrometer (JASCO Corp., Tokyo, Japan). JEOL EX-270 (270 MHz), JNM-LA 500 (500 MHz), and JNM-ECA 600 K (600 MHz) instruments (all JEOL Ltd.) were used for ^1^H-NMR and ^13^C-NMR measurements, and tetramethylsilane (TMS) was used as the internal standard. For HPLC, a LC-6AD pump, RID-10A differential refractive index detector, and SPD-10A UV–Vis detector were used (Shimadzu Corp.).

Silica Gel 60 N (Kanto Chemical Co., Inc., Tokyo, Japan) was used as the normal-phase adsorbent for column chromatography, and Chromatorex ODS DM 1020 T (Fuji Silicia; Fuji Silysia Chemica, Kasugai, Japan) was used as the reversed-phase system. For TLC, we used silica gel 60 F_254_ (normal phase; Merck KGaA, Darmstadt, Germany), RP-18 60 F_254_ (reversed phase; Merck KGaA), silica gel 60 F_254_ HPTLC (normal phase; Merck KGaA), and RP-18 WF_254_ HPTLC (reversed phase; Merck KGaA). Spot detection was carried out by UV irradiation (254 nm) and spraying 1% Ce(SO_4_)/10% H_2_SO_4_ aqueous solution followed by heating. Reagents (special grade) purchased from Wako Pure Chemical Industries, Ltd (Osaka, Japan). were used unless otherwise specified.

### Plant material

The seeds of *C. auriculata* cultivated in India were purchased from NTH India Pvt. Ltd. (Gurgaon, India) in 2009 and identified by one of the authors (MY). A voucher specimen is kept in our laboratory (KPU-N.T.H. CAS-1).

### Extraction and isolation

The seeds of *C. auriculata* (3929 g) were crushed and extracted three times with MeOH under reflux for 3 h. Evaporation of the solvent under reduced pressure provided a MeOH extract (430.6 g, 10.96%). A part of the MeOH extract (399.0 g) suspended in H_2_O was extracted with *n*-hexane to furnish an *n*-hexane-soluble fraction (46.4 g, 1.27%) and a H_2_O-soluble fraction (350.8 g, 9.63%). A part of the H_2_O-soluble fraction (322.7 g) was subjected to Diaion HP-20 column chromatography (H_2_O → MeOH) to give the H_2_O-eluted fraction (248.2 g, 7.41%) and the MeOH-eluted fraction (63.2 g, 1.89%), respectively. The MeOH-eluted fraction (60.0 g) was subjected to normal-phase silica gel column chromatography [1.5 kg, CHCl_3_ →  CHCl_3_:MeOH (20:1, v/v) → CHCl_3_:MeOH:H_2_O (10:3:1 → 7:3:1 → 6:4:1, v/v/v) → MeOH] to give nine fractions (Fr.) [ Fr. 1 (0.2 g), Fr. 2 (3.0 g), Fr. 3 (2.7 g), Fr. 4 (0.6 g), Fr. 5 (10.4 g), Fr. 6 (5.7 g), Fr. 7 (16.7 g), Fr. 8 (16.3 g), Fr. 9 (0.9 g)].

Fr. 5 (10.4 g) was further separated by ODS column chromatography [300 g, MeOH:H_2_O (20:80 → 30:70 → 40:60 → 50:50 → 60:40 → 70:30 → 80:20, v/v) → MeOH]  to  give 13 fractions [Fr. 5-1 to 5-4, Fr. 5-5 (1991 mg), Fr. 5-6 (588 mg), Fr. 5-7, Fr. 5-8 (609 mg), Fr. 5-9 (269 mg), Fr. 5-10 (820 mg), Fr. 5-11 to 5-13]. Fr. 5-5 (1991 mg) was separated and purified by HPLC [MeOH:H_2_O (45:55 and 40:60, v/v) or CH_3_CN:MeOH:H_2_O (19:8:73, v/v/v)] to give rumejaposide E (**3**, 6.6 mg, 0.00021%) and rumejaposide F (**4**, 6.1 mg, 0.00019%). Fr. 5-6 (588 mg) was also subjected to HPLC [MeOH:H_2_O (45:55, v/v)] to give rumejaposide E (**3**, 17.7 mg, 0.00056%) and rumejaposide F (**4**, 18.1 mg, 0.00057%). Fr. 5-8 (609 mg) was subjected to silica gel column chromatography [CHCl_3_ → CHCl_3_:MeOH (20:1, v/v) → CHCl_3_:MeOH:H_2_O (10:3:1, v/v/v, lower layer)] and HPLC [MeOH:H_2_O (32:68, v/v)] to give auriculataoside A (**1**, 240.4 mg, 0.0076%). Fr. 5-9 (269 mg) was separated by HPLC [MeOH:H_2_O (70:30, v/v)] to furnish auriculataoside A (**1**, 25.6 mg, 0.00081%) and auriculataoside B (**2**, 57.9 mg, 0.0018%). Fr. 5-10 (400 mg) was purified by HPLC [MeOH:H_2_O (75:25, v/v)] to obtain auriculataoside B (**2**, 142.0 mg, 0.0092%).

#### Auriculataoside A (**1**)

Red powder; [*α*]_D_^24^ – 129.5 (*c *= 0.20, 1,4-dioxane); UV (MeOH) *λ*_max_ (log ε) 225 (4.58), 273 (4.71), 399 (4.09), 433 (4.02) nm; CD (MeOH) nm (*Δε*) 217 (+ 49.0), 234 (− 17.2), 242 (−6.7), 270 (− 38.0), 284 (+ 25.5); IR (KBr) *v*_max_ 3450, 2948, 1655, 1070 cm^−1^; ^1^H-NMR (DMSO-*d*_6_, 500 MHz) 1.15, 2.24 (3H each, both s, C*H*_3_-3′, 3), 2.61 (2H, br s, H_2_-4′), 2.74, 2.88 (1H each, both d, *J  *= 16.8 Hz, H-2′), 3.21 (1H, m, H-4″), 3.35 (1H, m, H-3″), 3.43 (1H, m, H-5″), 3.47 (1H, m, H-2″), 3.48 (1H, m, H-6″a), 3.65, 3.89 (3H each, both s, OC*H*_3_-6′, 6), 3.74 (1H, dd, *J * = 11.2, 5.5 Hz, H-6″b), 4.79 (1H, br s, O*H*-3′), 5.04 (1H, d, *J * = 7.7 Hz, H-1″), 6.20, 6.88 (1H each, both d, *J * = 2.1 Hz, H-5′, 7′), 7.22, 7.59 (1H each, both br s, H-2, 4), 7.55 (1H, s, H-5), 11.88 (1H, br s, O*H*-1), 12.19 (1H, br s, O*H*-8), 19.20 (1H, br s, O*H*-9′); ^13^C-NMR data, see Table 1; positive-ion FAB-MS *m/z* 755 [M+Na]^+^; high-resolution (HR)FAB-MS: *m/z* 755.1958 (calculated for C_38_H_36_O_15_ [M+Na]^+^, 755.1952).

#### Auriculataoside B (**2**)

Red powder; [*α*]_D_^24^ – 41.3 (*c *= 0.28, 1,4-dioxane); UV (MeOH) *λ*_max_ (log ε) 225 (4.62), 273 (4.74), 399 (3.72), 433 (4.05) nm; CD (MeOH) nm (*Δε*) 217 (– 42.8), 233 (+ 20.5), 268 (+ 23.8), 282 (− 33.9); IR (KBr) *v*_max_ 3450, 2948, 1655, 1070 cm^−1^; ^1^H-NMR (DMSO-*d*_6_, 500 MHz) 1.18, 2.24 (3H each, both s, C*H*_3_-3′, 3), 2.57, 2.75 (1H each, both d, *J  *= 16.2 Hz, H_2_-4′), 2.72, 2.90 (1H each, both d, *J  *= 16.8 Hz, H_2_-2′), 3.22 (1H, m, H-4″), 3.35 (1H, m, H-3″), 3.44 (1H, m, H-5″), 3.47 (1H, m, H-2″), 3.50 (1H, m, H-6″a), 3.66, 3.85 (3H each, both s, OC*H*_3_-6′, 6), 3.75 (1H, dd, *J  *= 11.6, 5.5 Hz, H-6″b), 4.78 (1H, br s, O*H*-3′), 5.02 (1H, d, *J  *= 7.7 Hz, H-1″), 6.20, 6.89 (1H each, br s, H-5′, 7′), 7.18, 7.53 (1H each, both br s, H-2, 4), 7.52 (1H, s, H-5), 11.90 (1H, br s, O*H*-1), 12.27 (1H, br s, O*H*-8), 19.20 (1H, br s, O*H*-9′); ^13^C-NMR data, see Table 1; positive-ion FAB-MS *m/z* 755 [M+Na]^+^; HRFAB-MS: *m/z* 755.1952 (calculated for C_38_H_36_O_15_ [M+Na]^+^, 755.1952).

#### Acid hydrolysis and monosaccharide identification of** 1** and** 2**

Compounds **1** and **2** (1 mg each) were mixed with 1.0 M HCl (1.0 mL) and each solution was refluxed for 3 h. The reaction mixture was immersed in ice-cold water and neutralized with Amberlite IRA-400 (OH^−^ form), and the resin was removed by filtration. After extraction with EtOAc, the aqueous layer was analyzed by HPLC [HPLC column, Kaseisorb LC NH_2_-60-5, 4.6 mm i.d. × 250 mm (Tokyo Kasei Co., Ltd., Tokyo, Japan); detection: optical mobile phase, CH_3_CN–H_2_O (85:15, v/v); flow rate: 0.80 mL/min; column temperature: room temperature] equipped with an optical rotation detector (Shodex OR-2; Showa Denko K.K., Tokyo, Japan). d-glucose (from **1** and **2**) was confirmed by comparing its retention time (*t*_R_) and optical rotation with those of an authentic sample; *t*_R_ = 11.5 min (d-glucose, positive optical rotation).

### Bioassay methods

#### Reagents

Dulbecco’s modified Eagle’s medium (DMEM, 4500 mg/L glucose) was purchased from Sigma-Aldrich (St. Louis, MO, USA); fetal bovine serum (FBS). Penicillin, and streptomycin were purchased from Gibco (Invitrogen, Carlsbad, CA, USA). The tetrazolium salt MTT was obtained from Dojindo Laboratories (Kumamoto, Japan); Soluene-350 was obtained from PerkinElmer, Inc. (Waltham, MA, USA); Blocking One was from Nacalai Tesque (Kyoto, Japan); phosphatase inhibitor was purchased from Roche (Mannheim, Germany); proteinase inhibitor, the Protein Assay kit, and other chemicals were purchased from Wako Pure Chemical Industries, Ltd. Six- and 24-well multiwell plates and 96-well microplates were purchased from Greiner Japan (Tokyo, Japan).

#### Cell culture

Murine B16 melanoma 4A5 cells (RCB0557) were obtained from Riken Cell Bank (Tsukuba, Japan) and HDF (code no. CA10605n) were purchased from Toyobo Co., Ltd. (Tokyo, Japan). The B16 melanoma 4A5 cells were grown in DMEM (4500 mg/L glucose) medium (Sigma-Aldrich) containing 10% FBS, 100 units/mL penicillin, and 100 μg/mL streptomycin under conditions of 5% CO_2_ and 37 °C. The HDF cells were grown in DMEM (1000 mg/L glucose) medium. Cells were harvested by incubation in phosphate-buffered saline (PBS) containing 1 mM EDTA and 0.25% trypsin for approximately 5 min at 37 °C and were used for subsequent bioassays.

#### Effects on melanogenesis

##### Melanin production

The screening test for melanogenesis using B16 melanoma 4A5 cells was performed as described previously [23]. B16 melanoma 4A5 cells (2.0 × 10^4^ cells/400 μL/well) were seeded in a 24-well multiwell plate and pre-cultured for 24 h (5% CO_2_, 37 °C), following which the test sample and theophylline (as melanogenesis stimulator; final concentration 1 mM) were added. After culturing for 72 h, the cells were detached and recovered by trypsin treatment, washed with PBS, and dissolved in NaOH aq. (120 μL/well, 80 °C, 15 min). The cell lysate was fractionated (100 μL/well) in a 96-well microplate, and the absorbance of melanin produced was measured with a microplate reader (SH-1000; Corona Electric Co. Ltd., Hitachinaka, Japan) (wavelength 405 nm). The test sample was dissolved in DMSO and added to the medium (final concentration of DMSO 0.1%). Inhibition was measured as:

$${\text{Inhibition }}\left( {{\%}} \right) = \left\{ {\left[ {A{-}B /\left( {C/100} \right)} \right]/A} \right\} \, \times \,100$$

where *A* is the absorbance without test sample (control); *B* is the absorbance with test sample; *C* is cell viability (%) with test sample.

##### Cell viability

B16 melanoma 4A5 cells (5.0 × 10^3^ cells/100 μL/well) and HDF (5.0 × 10^3^ cells/100 μL/well) were seeded in a 96-well microplate and pre-cultured for 24 h (5% CO_2_, 37 °C), following which the test sample and theophylline (final concentration 1 mM) were added. After culturing for 71 h (5% CO_2_, 37 °C), 10 μL of MTT (0.5%) was added. After incubation for 1 h (5% CO_2_, 37 °C), the formazan present, which was produced as a catalytic product of MTT, was dissolved in 100 µL of 2-propanol containing 0.04 M HCl, and the absorbance was measured with a microplate reader (SH-1000; Corona Electric Co. Ltd.; measurement wavelength 570 nm, reference wavelength 655 nm). The test sample was dissolved in DMSO and added to the medium (final concentration of DMSO 0.1%). Viability of the cells was measured as:

$${\text{Viability }}\left( {{\%}} \right) \, = \, 1{-} \, \left[ {\left( {A{-}B} \right) \, / \, A} \right] \, \times \, 100$$

where *A* is the absorbance without test sample (control); *B* is the absorbance with test sample.

#### Effects on mushroom tyrosinase activity

Inhibitory effects on mushroom tyrosinase activity were examined as reported elsewhere [9, 31]. A phosphate buffer solution (pH 6.5) containing 2.5 mM L-DOPA was dispensed into a 96-well microplate in the amount of 70 μL/well, and 20 μL of test sample (DMSO solution) was added. Next, 120 μL/well of a phosphate buffer solution (80 units/mL) of mushroom-derived tyrosinase was added to each well to induce the enzymatic reaction (25 °C, 5 min), and the absorbance of the produced dopaquinone was measured using a microplate reader (SH-1000; Corona Electric Co. Ltd.) (measurement wavelength 405 nm). Inhibition was measured as:

$${\text{Inhibition }}\left( {{\%}} \right) \, = \, \left[ {\left( {A{-}B} \right) \, / \, A} \right] \, \times \, 100$$

where *A* is the absorbance without test sample (control); *B* is the absorbance with test sample.

#### Effects on melanoma tyrosinase activity

Inhibitory effects on melanoma tyrosinase activity were examined using methods described elsewhere, with slight modifications [31–33]. B16 melanoma 4A5 cells were cultured for 72 h, and the cells were then detached by trypsin treatment, collected, and washed with PBS. Then, 1 mL of 0.1% Triton X-100 in phosphate buffer solution (pH 6.5) was added. The cells were disrupted using an ultrasonic cell disruptor under cooling with ice and then centrifuged at 11,000 *g* for 10 min. The supernatant was used as the crude enzyme solution, and the amount of enzyme was quantified using an enzymatic reaction, as follows. Phosphate buffer solution (pH 6.5) containing 2.5 mM L-DOPA (80 μL/well) was dispensed into a 96-well microplate, and 10 μL of test substance (DMSO solution) was added. Next, crude tyrosinase solution was diluted with phosphate buffer solution (pH 6.5) to 10 µg/mL protein. The diluted enzyme solution was add to each well (10 μL/well), and the enzymatic reaction (37 °C) was carried out for 24 h. A microplate reader (SH-1000; Corona Electric Co., Ltd.) was used to measure the absorbance of melanin (measurement wavelength 405 nm). Inhibition was measured as:

$${\text{Inhibition }}\left( {{\%}} \right) \, = \, \left[ {\left( {A{-}B} \right) \, / \, A} \right] \, \times \, 100$$

where *A* is the absorbance without test sample (control); *B* is the absorbance with test sample.

#### Effects on autoxidation

The autoxidation of dopachrome was performed according to previously reported methods, with slight modifications [36, 37]. A phosphate buffer solution (125 µL) containing mushroom-derived tyrosinase (150 units/mL, pH 6.5) was pre-incubated at 25 °C for 10 min. Then, 125 μL of 0.03% L-DOPA phosphate buffer solution was added, and the incubation was carried on for a further 10 min. Next, 125 μL of test sample (DMSO solution) was added, and the reaction was allowed to proceed for 60 min (25 °C). To terminate the reaction, 50 μL of 1.0 M HCl solution was added, and the solution centrifuged conducted at 10,000 *g* for 15 min. After the residue was washed with ethanol, it was dissolved in 1 mL of Soluene-350 (60 °C), and the absorbance of the melanin produced was measured using a microplate reader (SH-1000; Corona Electric Co. Ltd.) (wavelength 405 nm). Inhibition was measured as:

$${\text{Inhibition }}\left( {{\%}} \right){ = }\left[ {\left( {A{-}B} \right) \, / \, A} \right] \, \times \, 100$$

where *A* is the absorbance without test sample (control); *B* is the absorbance with test sample.

#### Effects on MITF, tyrosinase, TRP-1, and TRP-2 protein levels

B16 melanoma 4A5 cells (1.0 × 10^5^ cells/2 mL/well) were seeded in a 6-well multiwell plate and pre-incubated for 24 h, following which the test sample and theophylline (final concentration 1 mM) were added and the cell solution incubated for a further 72 h. Then, 140 μL of lysis buffer was added after washing with PBS. Cells detached with a cell scraper were disrupted with an ultrasonic cell disruptor under cooling with ice to extract the protein. The supernatant was centrifuged at 11,000 *g* for 10 min. The amount of protein was determined by the Lowry method and denatured at 100 °C for 5 min.

An aliquot of extracted protein (20 μg/lane) was electrophoresed on 10% sodium dodecyl sulfate-polyacrylamide gels for 1 h, washed with Tris-buffered saline (T-TBS), and transferred to a membrane (PVDF membrane) using a transfer device. After washing with T-TBS, blocking was carried out with Blocking One (Nacalai Tesque, Inc., Kyoto, Japan) for 1 h, and incubation was commenced with primary antibody against each protein in 5% Blocking One/T-TBS solution (0.2 μg/mL) for 1 h. After washing with T-TBS, the membrane was incubated with horseradish peroxidase-conjugated secondary antibody in 5% Blocking One/T-TBS solution (0.3 μg/mL) for 1 h. Immunoreactive proteins were detected using a chemiluminescence kit (Chemi-Lumi One; Nacalai Tesque) according to the manufacturer’s instructions. Each target band was measured by a lumino image analyzer (LAS-4000 mini; Fujifilm, Tokyo Japan); Multi Gauge V3.0 software was used to analyze the data (Fujifilm).

The primary antibody was diluted 1000-fold. Anti-tyrosinase (goat), anti-TRP-1 (rabbit), and anti-TRP-2 (rabbit) were purchased from Santa Cruz Biotechnology (Dallas, TX, USA). Anti-MITF (rabbit) and anti-β-actin (rabbit) were from Cell Signaling Technology (Tokyo, Japan). The secondary antibody was diluted 4000-fold. Anti-rabbit antibody was from Cell Signaling Technology and rabbit anti-goat antibody was from Thermo Fisher Scientific (Waltham, MA, USA).

### Statistical analysis

Values were expressed as the mean ± standard error of the mean. A one-way analysis of variance followed by Dunnett’s test was used to determine the statistical significance of the differences between the control group and test sample-treated groups. *P* values of < 0.05 were regarded as significant.

## **Electronic supplementary material**

**Below is the link to the electronic supplementary material.**

**Supplementary material 1 (DOCX 533** **kb)**
