# Correction to: Inhibition of melanin production by anthracenone dimer glycosides isolated from *Cassia auriculate* seeds

**DOI:** 10.1007/s11418-020-01402-z

**Published:** 2020-04-10

**Authors:** Weicheng Wang, Yi Zhang, Souichi Nakashima, Seikou Nakamura, Tao Wang, Masayuki Yoshikawa, Hisashi Matsuda

**Affiliations:** 1 Kyoto Pharmaceutical University, Misasagi, Yamashina-ku, Kyoto, 607-8412 Japan; 2 Tianjin State Key Laboratory of Modern Chinese Medicine, Tianjin University of Traditional Chinese Medicine, 312 Anshanxi Road, Nankai District, Tianjin, 300193 China; 3 N.T.H Co., Ltd., 4F Sky-ebisu Bldg, 1-8-11 Ebisu, Shibuya-ku, Tokyo, 150-0013 Japan

## Correction to: Journal of Natural Medicines (2019) 73:439–449 https://doi.org/10.1007/s11418-018-01276-2

The article Inhibition of melanin production by anthracenone dimer glycosides isolated from *Cassia auriculata* seeds, written by Weicheng Wang, Yi Zhang, Souichi Nakashima, Seikou Nakamura, Tao Wang, Masayuki Yoshikawa and Hisashi Matsuda was originally published Online First without Open Access. After publication in volume 73 issue 3, page 439–449 the author decided to opt for Open Choice and to make the article an Open Access publication. Therefore, the copyright of the article has been changed to © The Author(s) 2020 and the article is forthwith distributed under the terms of the Creative Commons Attribution 4.0 International License (https://creativecommons.org/licenses/by/4.0/), which permits use, duplication, adaptation, distribution and reproduction in any medium or format, as long as you give appropriate credit to the original author(s) and the source, provide a link to the Creative Commons license, and indicate if changes were made.

The original article was updated.

