# Supplementary material for: Inhibition of melanin production by anthracenone dimer glycosides isolated from Cassia auriculata seeds
Source: J Nat Med. 2019 Mar 7;73(3):439–49. doi: 10.1007/s11418-018-01276-2 (PMC7176596; doi:10.1007/s11418-018-01276-2)
Supplement: Supplementary file 1 — Supplementary material 1 (DOCX 533 kb) [file 11418_2018_1276_MOESM1_ESM.docx]

**Supplementary material**

**Inhibition of Melanin Production by Anthracenone Dimer Glycosides**

**Isolated from *Cassia auriculata* Seeds**

Weicheng Wang^1^, Yi Zhang^1,2^, Souichi Nakashima^1,3^, Seikou Nakamura^1^, Tao Wang^1,2^,

Masayuki Yoshikawa^1^, and Hisashi Matsuda^1^

^1^ Kyoto Pharmaceutical University; Misasagi, Yamashina-ku, Kyoto 607–8412, Japan

^2^ Tianjin State Key Laboratory of Modern Chinese Medicine, Tianjin University of Traditional Chinese Medic ine; 312 Anshanxi Road, Nankai District, Tianjin 300193, China

^3^ N.T.H Co., Ltd.; 4F Sky-ebisu Bldg, 1–8–11 Ebisu, Shibuya-ku, Tokyo 150–0013, Japan.

**Fig. S1** Structure of (3'S, P)-anhydrophlegmacin-9,10-quinone 8'-O-methyl ether (**S1**) [24]

auriculataoside A (**1**) auriculataoside B (**2**)


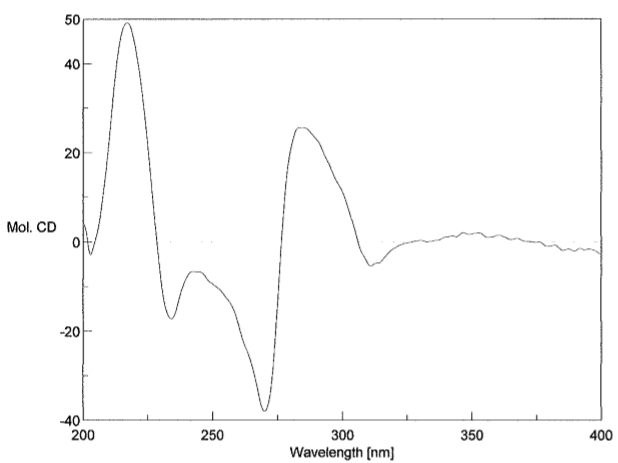

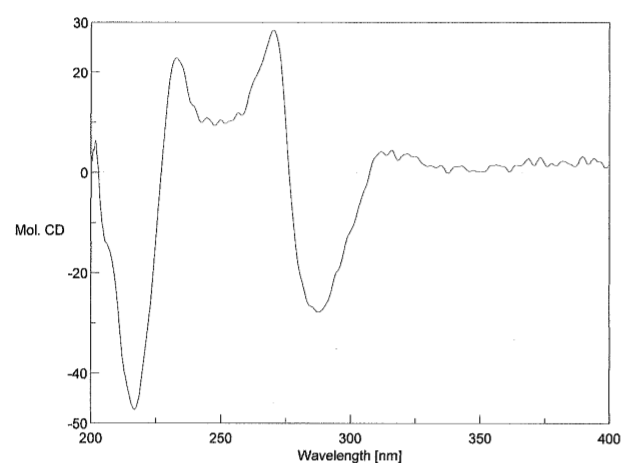


**Fig. S2** CD spectra (MeOH) of auriculataosides A (**1**) and B (**2**)


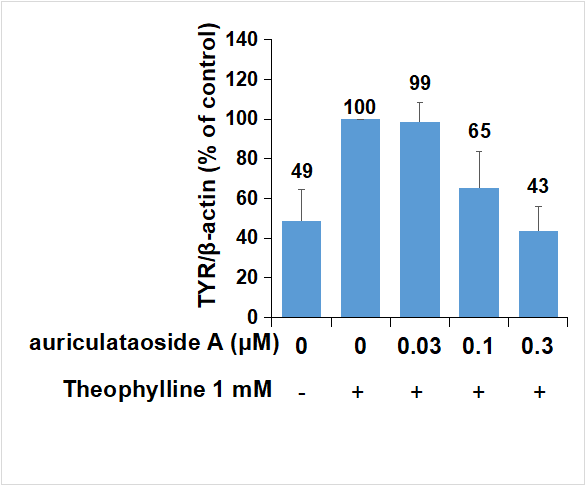

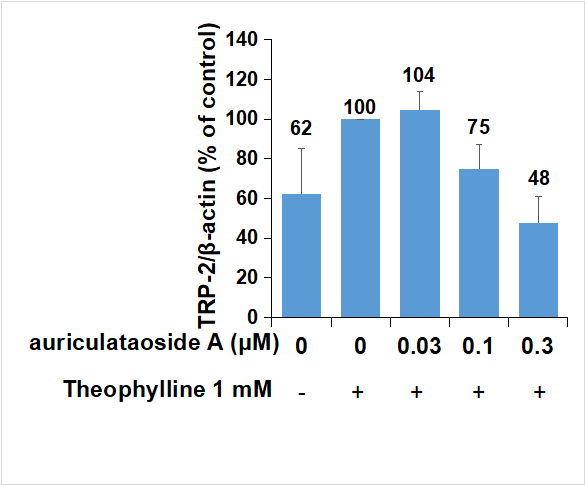

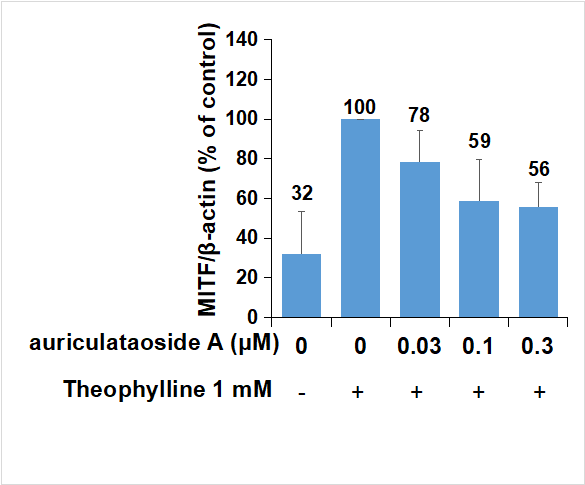

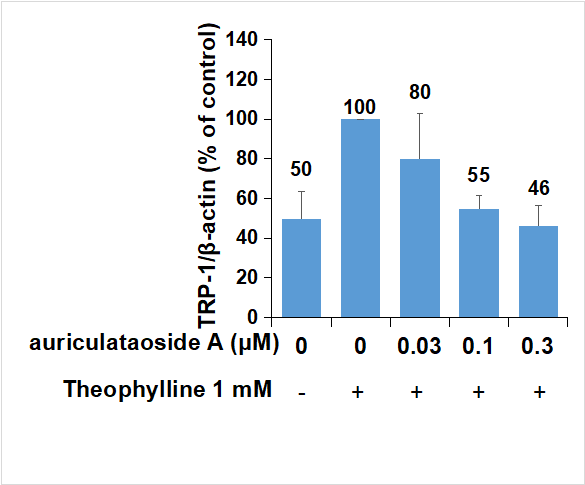

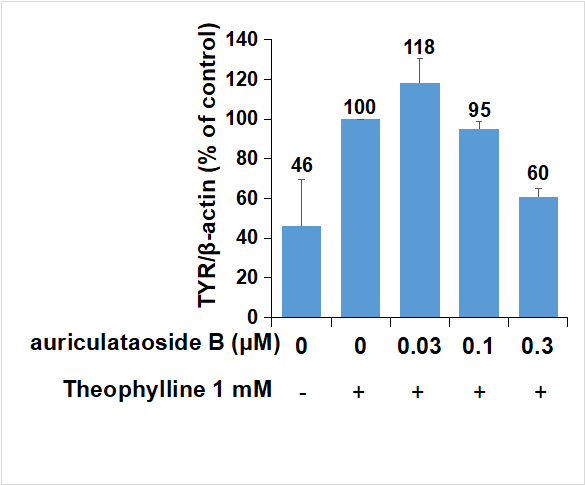

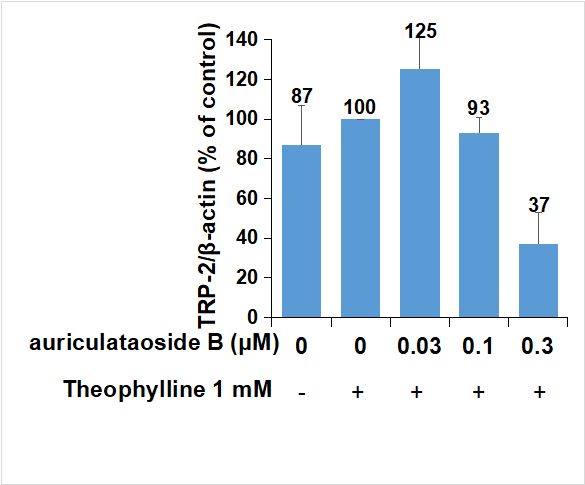

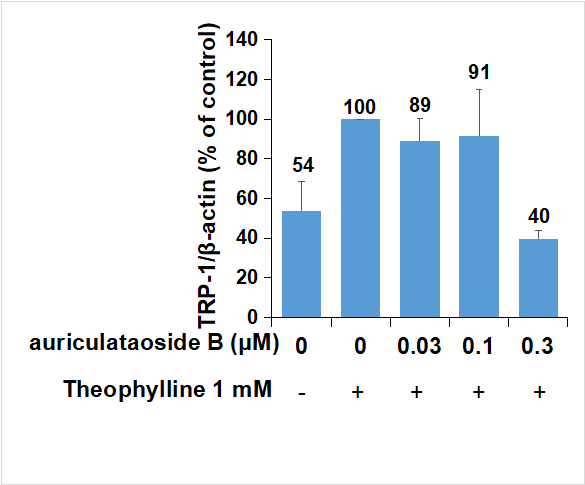

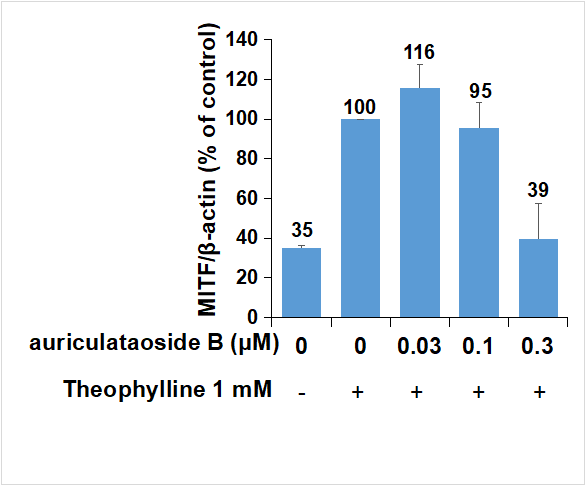


**Fig. S3** Effects of auriculataosides A (**1**) and B (**2**) on MITF, TYR, TRP-1 and TRP-2 protein levels

The target/β-actin in control group [theophylline (+), test sample (-)] was calculated to be 100%, and those in the test sample-treated group were expressed as % of control. Each bar represents the mean with S.E.M. (N=3).
